# Genome-Wide Identification, Expression and Evolution Analysis of m6A Writers, Readers and Erasers in *Aegilops_tauschii*

**DOI:** 10.3390/plants12142747

**Published:** 2023-07-24

**Authors:** Huiyuan Lin, Tingrui Shi, Ying Zhang, Chuyang He, Qiying Zhang, Zhiping Mo, Wenqiu Pan, Xiaojun Nie

**Affiliations:** 1 State Key Laboratory of Crop Stress Biology in Arid Areas, College of Agronomy and Yangling Branch of China Wheat Improvement Center, Northwest A&F University, Yangling 712100, Chinashitingrui@nwafu.edu.cn (T.S.);; 2 Australia-China Joint Research Centre for Abiotic and Biotic Stress Management in Agriculture, Horticulture and Forestry, Yangling 712100, China

**Keywords:** m6A, expression profile, genetic variations, *Aegilops tauschii*

## Abstract

N6-methyladenosine modifications (m6A) is one of the most abundant and prevalent post-transcriptional RNA modifications in plants, playing the crucial role in plant growth and development and stress adaptation. However, the m6A regulatory machinery in *Aegilops_tauschii*, the D genome progenitor of common wheat, is not well understood at present. Here, we systematically identified the m6A-related genes in *Aegilops* with a genome-wide search approach. In total, 25 putative m6A genes composed of 5 writers, 13 readers and 7 erasers were obtained. A phylogenetic analysis clearly grouped them into three subfamilies with the same subfamily showing similar gene structures and conserved domains. These m6A genes were found to contain a large number of cis-acting elements associating with plant hormones, regulation of growth and development as well as stress response, suggesting their widespread regulation function. Furthermore, the expression profiling of them was investigated using RNA-seq data to obtain stress-responsive candidates, of which 5 were further validated with a qPCR analysis. Finally, the genetic variation of m6A-related genes was investigated between Aegilops and D subgenome of wheat based on re-sequencing data, and an obvious genetic bottleneck occurred on them during the wheat domestication process. The promising haplotype association with domestication and agronomic traits was also detected. This study provided some insights on the genomic organization and evolutionary features of m6A-related genes in Aegilops, which will facilitate the further functional study and also contribute to broaden the genetic basis for genetic improvement in wheat and other crops.

## 1. Introduction

More than 160 RNA modifications have been identified in eukaryotes, including N6-adenylate methylation (m6A), N1-adenylate methylation (m1A), cytosine hydroxylation (m5C), etc. [1,2,3]. Among them, m6A is the most abundant and prevalent modification, which is widely found in mRNA, tRNA, miRNA and long non-coding RNA [4]. The m6A regulatory machinery is also conserved in different organisms, consisting of methyltransferase complex (writer), demethylases (eraser) and m6A binding proteins (reader). It is a dynamic and reversible process that is catalyzed by the transformation of the methyl group from the methyl donor SAM to the sixth nitrogen atom of adenine base through the methyltransferase complex, and it can also be erased by demethylase (Eraser). At the same time, m6A readers play the role in binding on the modified site to read the methylation modification to achieve regulatory function [5].

As a new hotspot in the field of epitranscriptome, extensive studies have been performed to demonstrate the crucial and indispensable role of m6A playing in diverse biological processes. It has been shown that m6A had a regulatory function on selective shearing of pre-mRNA and processing of mRNA at the post-transcriptional level and then affected mRNA stability, translation, secondary structure and transport. Currently, most of the m6A-related studies were conducted in animals, especially in human [6,7]. With the advancement of detection technology, the related studies have gradually been performed in plants, mainly in model species, such as Arabidopsis and rice [8]. Based on the homology-search method, a total of 5, 13 and 13 m6A writers, readers and erasers were found in Arabidopsis, respectively [9,10]. A further functional analysis demonstrated that disruption of the key m6A methyltransferase MTA leads to embryonic death, as do deletions of the other m6A writer core components MTB, FIP37 and VIR in Arabidopsis [11]. Duan et al. found that ALKBH10B regulated the flowering transition process of Arabidopsis by mediating demethylation modification of flowering related genes [12]. Through MeRIP-seq analysis, more than 1000 genes could be induced by ALKBH10B to display differential methylation. Meanwhile, a transcriptome-wide profiling analysis revealed a unique distribution of m6A in Arabidopsis, and a number of plant-specific signaling pathways associated with photosynthesis were found to be affected by m6A [13]. These results demonstrated that m6A played an important role in plant growth and development. Some studies in other non-model plants are gradually performed. In poplar, the overexpression of PtrMTA significantly increased the trichome density and promoted root system development and then showed more tolerance to drought stress [14]. An m6A modification is also involved in regulating salt tolerance in sweet sorghum [15], controlling cadmium stress adaptation in barley as well as responding to it in wheat [16]. Taken together, m6A plays an important regulatory role in plant growth and development and response to abiotic stresses.

*Aegilops tauschii*, as the donor of D subgenomes of common wheat, is considered to be the important genetic reservoir and gene pool for wheat genetic improvement and exhibits formidable vitality and strong growth potential, capable of thriving in arid deserts, saline soils and mud flats. With the change of the global climate, the distribution range of *Aegilops tauschii* has a trend of further expansion, suggesting its abundant genetic diversity regarding resistance to stress, insects and disease [17]. A comprehensive and systematic analysis of the genetic diversity of *Aegilops tauschii* can not only provide information for the study of the origin and evolution of wheat, but also furnish reliable data support for the utilization of its superior agronomic traits and disease resistance genes. However, studies on the m6A genes in *Aegilops tauschii* are scarce up to now, and their biological function remains to be explored [18]. Identification of gene family at the whole-genome level has become feasible in *Aegilops tauschii* with the completion of its reference genome recently. In this study, we performed a genome-wide search of m6A-related genes in *Aegilops tauschii*, and then, the phylogenetic relationship and chromosome localization were investigated. Furthermore, the salt-responsive and drought-responsive m6A genes were characterized based on RNA-seq data, and five of which were further validated using QPCR analysis. Finally, the genetic variations and haplotype analysis of the m6A genes in *Aegilops tauschii* and common wheat were investigated. This is the first study to identify m6A gene family in *Aegilops tauschii*, which provided the targets for a further function analysis to elucidate the mechanisms underlying stress tolerance in *Aegilops tauschii* and also contribute to better understand the evolution of this family in wheat and other crops.

## 2. Results

### 2.1. Genome-Wide Identification of m6A-Related Genes in Aegilops tauschii

**Figure 1:**
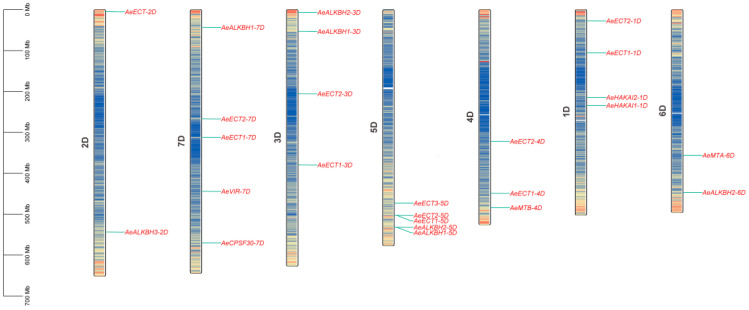
Chromosomal localization of m6A genes in *Aegilops tauschii*.

Through a genome-wide search, a total of 25 m6A-related genes were detected in *Aegilops tauschii genome*, including 5 m6A writers (2 AeMTs, 1 AeVIRs and 2 AeHAKAIs), 13 m6A readers (12 TdECTs and 1 TdCPSF30s), 7 m6A erasers (7 TdALKBHs) (Table 1 and Table 2). Each class displayed the distinct conserved domains that supported the prediction accuracy. Chromosome location analysis found that these 25 m6A-related genes were unevenly distributed on all of the 7 chromosomes (Figure 1), with Chr_7D and 5D having 5 m6A-related genes while Chr_2D and 6D possessing 2 m6A-related genes, ranked as the most and least abundant ones, respectively. It is found that the number of m6A genes was irrelevant with the chromosome size in *Aegilops tauschii* genome. Furthermore, their protein characteristic were also investigated. The size of them ranged from 267 (*AeALKBH2-6D*) to 2241 (*AeVIR-7D*) amid acid (AA) with the average of 655 AA. The molecular weights (MWs) of them ranged from 29.27 to 246.61 kDa with the average of 7.19 kDa. The isoelectric point (pI) ranged from 5.11 to 9.18 with the average of 7.16. Meanwhile, subcellular localization prediction found that they were located in the nucleus, chloroplast and other organelles of cells (Table 1), which was consistent with previous studies [19,20].

### 2.2. Phylogenetic Relationship and Sequence Characteristics Analysis

**Figure 2:**
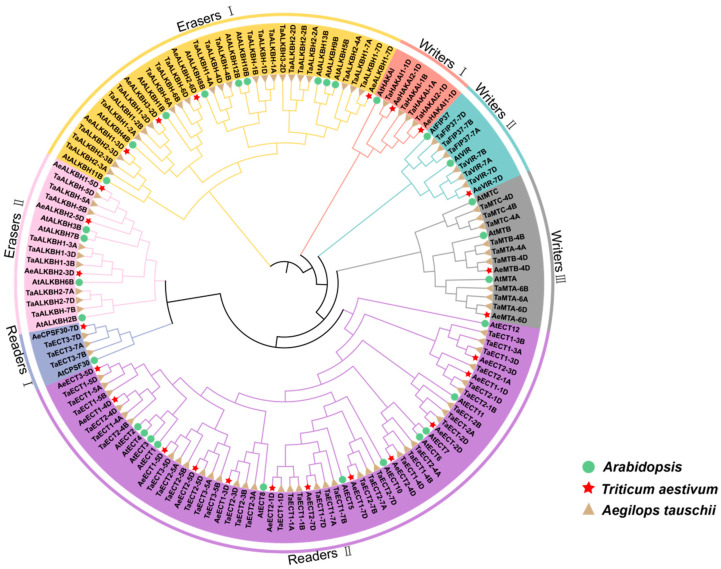
Phylogenetic tree of m6A genes in Triticum aestivum, Arabidopsis and *Aegilops tauschii*. The phylogenetic trees were constructed using MEGA 11.0 by the neighbor-joining (NJ) method with 1000 bootstrap replicates.

We further investigated the phylogenetic relationship and evolutionary relationships of these m6A-related proteins through constructing the phylogenetic tree using 35 Arabidopsis m6A-related gene proteins, 88 in wheat and 25 in *Aegilops tauschii*. Results exhibited that all these m6A-related genes could be divided into 3 groups according to the phylogenetic relationship, namely writers, readers, and erasers family (Figure 2). The writers family in *Aegilops tauschii* could be further classified into two subfamilies, namely ECT and CPSF30 groups. The readers family in *Aegilops tauschii* could be divided into four subgroups, corresponding to MT, FIP37, VIR and HAKAI proteins. The erasers family in *Aegilops tauschii* contains only ALKBH proteins. Each family contained the m6A genes from wheat, Arabidopsis and Aegilops, and no obvious deletion event was found, indicating that m6A-related proteins were evolutionarily conservative and their genetic differentiation occurred before the divergence of monocotyledon and dicot species.

**Figure 3:**
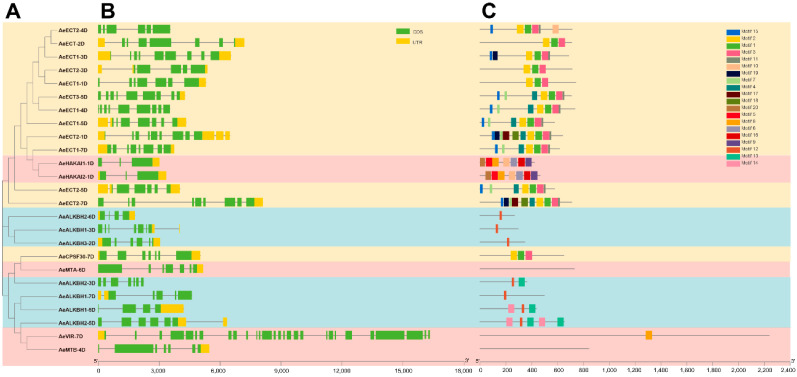
Phylogenetic tree, (**A**) exon–intron structures (**B**) and conserved protein motifs (**C**) of the m6A-related genes in *Aegilops tauschii*. The m6A regulatory components in phylogentic tress are marked by different colors and the different protein motifs are represented by different color boxes.

Furthermore, a total of 20 conserved motifs were identified in these Aetm6As (Figure 3C). It is recognized that all readers contained motif 1, motif 2 and motif 3, and these three motifs appeared consecutively in the same order. Motif 5 was identified in most erasers and motif 16 was identified in most writers, suggesting they were the conserved domain for the eraser and writer family. Obviously, the proteins with closer phylogenetic relationship shared more similar conserved motif organization. According to the PFAM annotation, motif 1, 2, 3 and 4 are the YTH domain, and motif 6, 17, 18, 19 and 20 are the Alkb domain. At the same time, AeMTA-6D and AeMTB-4D did not contain any conserved motif. Some motifs seemed to be group-specific, which might be linked with their specific biological functions. Finally, the exon–intron structure of them was also investigated. The exon numbers ranged from 3 to 28 with the majority of the 18 m6A genes having exon numbers between 6 and 9 (Figure 3B), suggesting they had rather complex gene structures. Like the conserved motif, the proteins with closer phylogenetic relationship shared more similar exon–intron organization and the different subfamily displayed a significantly different gene structure. Among them, the writers family had the most great sequence length variations with the most complex gene structures. Interestingly, the more motifs the genes contained, the more complex gene structures they showed.

### 2.3. Cis-Acting Element Analysis

**Figure 4:**
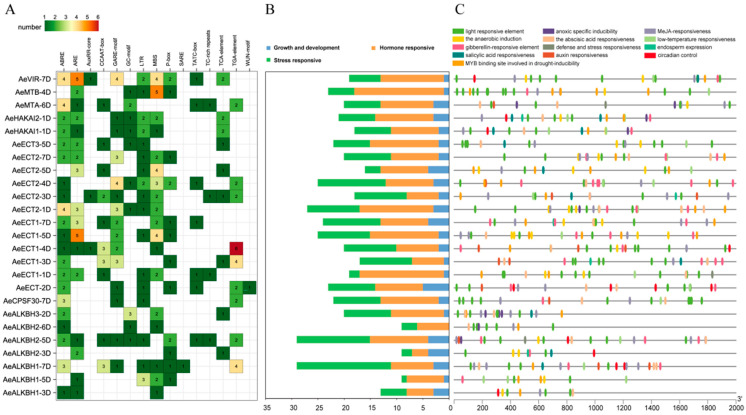
Cis-regulatory elements, the m6A-related genes in *Aegilops tauschii*. (**A**) The stress response-related elements and phytohormone-related elements in the promoter regions of each m6A-related genes; (**B**) Proportion of different types of cis-acting elements in the m6A-related genes; (**C**) All cis-acting elements found in m6A-related genes.

Cis-acting elements play an important role in the regulation of gene transcription and expression. A total of 99 cis-acting elements were identified in the promoter regions of Aetm6As, which were widely involved in biological growth and development, hormonal response, light response, metabolic regulation and stress response (Figure 4 and Table S1). Among them, the most abundant cis-acting elements were those responsive to light regulation, including sp1, G-box and I-box, which were found in all 25 Aetm6As. Meanwhile, 23 m6A genes contained G-box and 12 genes contained sp1, indicating that m6A genes may play important roles in controlling environmental adaptation. Furthermore, numerous hormone-related elements (Figure 4B), such as 9 Aetm6As contained auxin response elements (TGA-element, AuxRR-core), 20 Aetm6As contained abscisic acid response elements (ABRE), 19 Aetm6As contains gibberellin response element (TATC motif and P-box, GARE-motif), 22 Aetm6As contained methyl jasmonic acid response element (TGACG motif, CGTCA motif) and 9 Aetm6As contained salicylic acid response elements (TCA-element). Furthermore, the ABA-response element ABRE, the GA-response elements P-box and GARE-motif were found in 21 Aetm6As, indicating that m6A genes may be involved in the regulation of ABA and GA metabolism. Furthermore, drought-related elements (MBS) were found in 18 Aetm6As, implying that m6A genes may play an important role in drought stress response (Figure 4C).

### 2.4. Expressional Analysis of These Aetm6As under Drought and Salt Stresses

**Figure 5:**
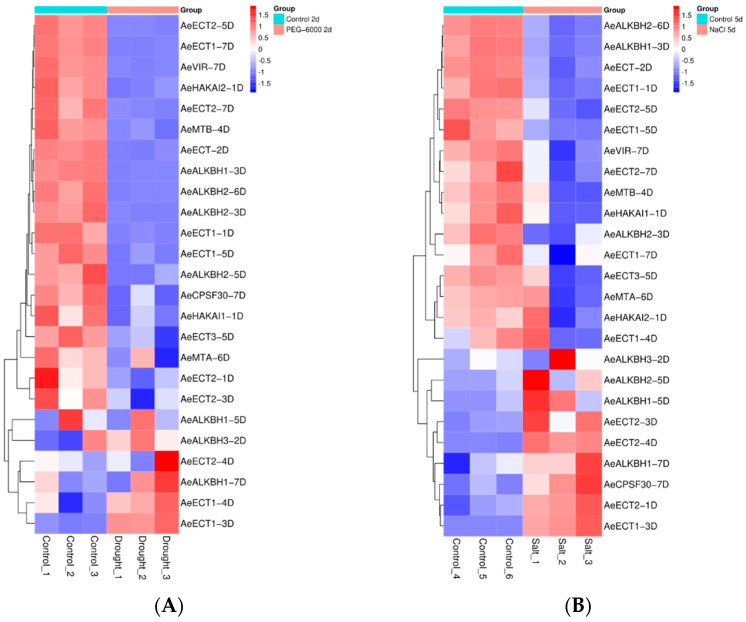
Expression profiles of m6A genes in *Aegilops tauschii* under drought (**A**) and salt (**B**) stresses.

In order to elucidate the function of Aetm6As in mediating drought and salt stress tolerance, we investigated their expression patterns under drought and salt stress based on RNA-seq data (Figure 5 and Table S2). The results showed that after two days of drought stress, nine m6A genes were down-regulated and five up-regulated, among which *AeECT2-5D* was up-regulated by 14-fold and *AeECT1-3D* was down-regulated by 18-fold. Meanwhile, after five days of salt stress, four m6A genes were up-regulated and five down-regulated, among which *AeECT1-5D* was up-regulated by 10-fold and *AeECT1-3D* was down-regulated by 10-fold. Taken together, it was found that the expressions of *AeALKBH2-6D*, *AeALKBH2-3D* and *AeALKBH2-1D* were all significantly down-regulated while AetECT1-3D was significantly up-regulated under both salt and drought stresses. On the contrary, *AeALKBH3-2D* and *AeALKBH1-5D* as well as *AeECT2-1D*, *AeECT2-3D* and *AeECT2-4D* displayed significantly higher expression under drought stress as compared to salt stress. These results suggested that m6A genes participated in the response to salt and drought stresses while different members exhibited functional differentiation. GO enrichment analysis of the differential expressed genes found that they significantly enriched into the “methylation” GO term (Figure S1 and Table S3), suggesting that the identified m6A genes are closely related to RNA methylation.

### 2.5. Genetic Variations and Haplotype of m6A-Related Genes in Aegilops tauschii and Wheat

**Figure 6:**
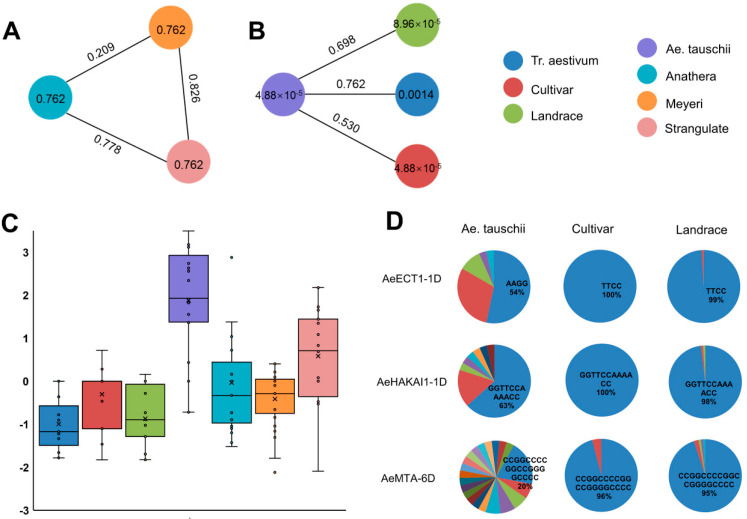
Analysis of genetic variation and haplotype of m6A gene in *Aegilops tauschii* and *Triticum aestivum.* L. (**A**) Comparison of Fst and nucleotide diversity (π) between subspecies of *Aegilops tauschii*. (**B**) Comparison of Fst and π values of *Aegilops tauschii* and *Triticum aestivum*, cultispecies and landrace. (**C**) Comparison of Tajima’s D values of *Aegilops tauschii*, subspecies of *Aegilops tauschii*, *Triticum aestivum*, cultispecies and landrace. (**D**) Haplotype statistics of m6A gene in *Aegilops tauschii*, cultispecies and landrace.

Based on the re-sequencing variome data, we identified the genetic diversity and haplotype frequency of each Aetm6A gene in Aegilops and common wheat populations. A total of 1062 SNP loci were found in the Aetm6A genes. Of these, 23 Aetm6A showed genetic variation and the remaining 2 genes had no SNPs. Nucleotide diversity (π), population divergence (Fst) and Tajima’D values of each m6A genes in different populations were calculated separately to assess the purifying selection on m6A genes during wheat evolution. The average value of Tajima’D in wheat landrace, wheat variety and *Ae. tauschii* was 0.8358944, 0.2786044 and −0.99409092 (Figure 6C and Table S4), suggesting that population size tend to contract or subject to balanced selection during wheat domestication. A significant reduction in genetic diversity (π) has been observed between wheat variety (0.000049) and *Ae. tauschii* (0.0013) (Figure 6A,B and Table S5). During *Ae tauschii* domesticated into bread wheat, the genetic variation of m6A genes reduced more than 10 times, suggesting that an obvious genetic bottleneck occurred between them. Subsequently, gene flow and genetic divergence between the wheat and *Ae. tauschii* were also detected. The Fst value between *Ae. tauschii* and wheat variety was 0.52 and indicates that there was a serious population divergence between wild and cultivated accessions. These results provided the useful information about wheat domestication and evolution from the perspective of m6A gene family.

It is obviously to find that all of the 23 m6A genes had more haplotypes in Aegilops compared to wheat, which was consistent with more genetic diversity in Aetm6A genes. At the same time, they also displayed significantly different haplotype frequency that Aegilops possessed many haplotypes with the main haplotype accounting for low proportion while wheat population had less haplotypes with the obvious main haplotype accounting for high proportion, suggesting the main haplotype was selected to increase its frequency in during wheat domestication. For example, the major haplotype of the *AeHAKAI2-1D* was CCTTAAGGAAGGGGAAGGAA, occupied 23% in Aegilops and increased to 96% in bread wheat. The major haplotype of the AeECT1-1D was AAGG in Aegilops with a 70% proportion, which was replaced by TTCC (100%) in bread wheat (Figure 6D and Table S6). The results indicate that wheat genetic diversity has been reduced and some specific haplotypes have been selected and fixed due to the behavior of artificial selection in wheat genetic improvement [21].

### 2.6. Validation of Salt-Responsive Aem6As through qRT-PCR Analysis

**Figure 7:**
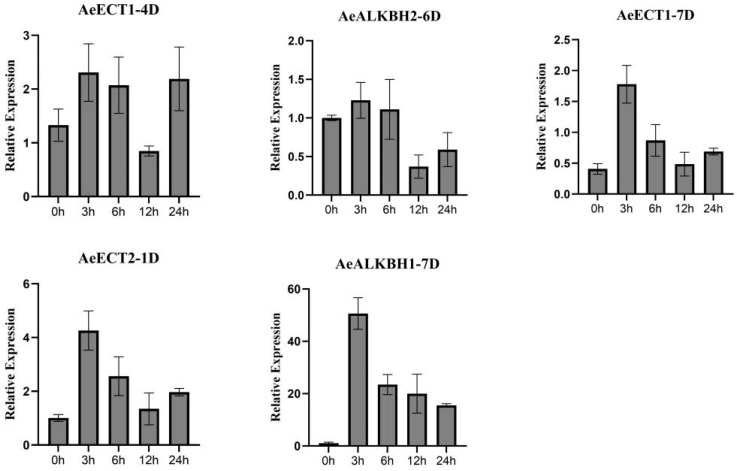
Relative expression levels of 5 randomly selected Aem6As genes under salt stress based on qRT-PCR analysis. The relative expression levels were determined by 2^−ΔΔCt^ method.

To further validate the salt-responsive candidates, we randomly selected 5 Aem6As, including AeECT1-4D, AeECT1-7D, AeECT2-1D, AeALKBH2-6D, AeALKBH1-7D to verify their expression patterns under salt stress (Figure 7). The results showed that the expression trends of these selected genes showed good consistence with those from an RNA-seq analysis, although they displayed completely diverse expression profiles under salt stress. It is found that all selected genes displayed up-regulated expression in response to salt stress with the highest expression at 3h, and then decreased gradually as time went by. Among them, AeALKBH1-7D showed significantly continuous high expression under salt stress, which might be considered as the potential targets for a functional study. The diverse expression patterns of these Aem6As implied their different roles underlying salt stress response.

## 3. Discussion

An m6A modification is one of the most abundant and prevalent post-transcriptional regulation mechanisms in organisms, playing important roles in regulating plant growth and development as well as biotic and abiotic stress response [2]. The systematic identification and characterization of m6A-related genes at the genome level not only sheds light on the genetic organization and evolution of the m6A regulatory machine, but also provided candidates for future functional studies, which have been conducted in Arabidopsis, rice and wheat [4,19]. In this study, we identified 25 m6A-related genes in *Aegilops tauschii* with a genome-wide search method, including 5 writers, 7 erasers and 13 readers, which is less than that of Arabidopsis (35) [22] and rice (35) [23]. No obvious duplication occurred in m6A genes in Aegilops. A sequence analysis showed these Aetm6As had some variations in the CDS length, exon number, protein sequence length, molecular weight, isoelectric point and subcellular localization. A total of 30 cis-acting elements were found in the promoter regions of Aetm6As, which were involved in plant hormone responses, plant developmental regulation as well as biotic and abiotic stresses. It is known that cis-acting elements played the crucial regulatory roles in influencing gene transcription and expression to control development and answer environmental stimuli in plants [24,25,26]. The diverse cis-acting elements in these Aetm6A genes indicated they played importance roles in regulating growth and development as well as stress response. We found that the abscisic acid response element ABRE was possessed by 21 m6A genes, indicating that m6A-related genes may be mainly involved in the control of ABA metabolism to regulate diverse biological processes, which was consistent with previous study [24]. 

Furthermore, the expression patterns of them under salt stress and drought stress were investigated and some stress-responsive candidates were obtained. The results show that *AeECT1-3D* was significantly up-regulated while *AeALKBH2-6D*, *AeALKBH2-3D* and *AeALKBH2-1D* were significantly down-regulated under both stress conditions, indicating their important roles in stress response and regulation. The results of qPCR also demonstrated that they played a role in response to salt stress. The identified stress-responsive candidates provided the candidates for future functional study. Using the resequencing data, the genetic variations of m6A-related genes were further investigated in Aegilops and bread wheat populations. A comparison of the haplotypes and genetic variation in Aegilops and wheat landrace as well as variety indicated that there was significant genetic divergence in m6A-related genes across all of these three populations, which was consistent with previous studies in NAC family and PYL gene families [27,28]. An obvious genetic bottleneck occurred when Aegilops domesticated into wheat, which provided some insight into the wheat evolution and domestication from the perspective of m6A gene family.

## 4. Materials and Methods

### 4.1. Genome-Wide Identification of m6A-Related Family in Aegilops tauschii

The whole genome reference and annotated protein sequences of *Aegilops tauschii* were downloaded from EnsemblPlants database (https://plants.ensembl.org/index.html, accessed on 30 November 2022) and then used as the local protein database. The Hidden Markov Model (HMM) profile of the m6A regulators domains (including PF05063, PF17098 and PF15912) were downloaded from PFAM database (http://pfam.xfam.org/ (accessed on 10 March 2023)) and used as the query to search against local protein database using the HMMER 3.0 with the threshold of E-value < 1 × 10^−5^ [29]. Meanwhile, the m6A regulatory proteins of Arabidopsis thaliana and rice were further used as queries to perform BLASTP search against the local protein database (E value < 1 × 10^−5^, agreement 50%, coverage > 50%) [30]. The results obtained from above two methods were integrated and removed the redundant manually to identify the putative m6A-related proteins in *Aegilops tauschii* (Aetm6As). Finally, these putative Aetm6As were submitted to the PFAM (https://pfam.xfam.org (accessed on 10 March 2023)) database and the NCBI-CDD (http://www.ncbi.nlm.nih.gov/cdd/ (accessed on 10 March 2023)) database to confirm the presence and completeness of the conserved domains. Only genes containing conserved and typical domains of m6A writers, erasers and readers were retained as candidates for further analysis. The EXPASy online software (https://www.expasy.org/ (accessed on 10 March 2023)) was used to predict the molecular weight(Mw), length of amino acid(AA) and isoelectric point(pI) of these m6A proteins were investigated. Their subcellular localization was predicted based on the Plant—mPLoc subcellular location (http://www.csbio.sjtu.edu.cn/bioinf/plant-multi/ (accessed on 10 March 2023)) and the Busca online software.

### 4.2. Phylogenetic and Sequence Characteristics Analysis

Multiple sequence alignment of the m6A-related proteins from *Aegilops tauschii*, Arabidopsis and *Triticum aestivum* were performed using the Clustalw tool [31]. The neighbor-joining (NJ) method embedded in MEGA 11.0 software [32] was used to construct phylogenetic tree with the Boostrap parameter set to 1000 and then was visualized with iTOL (https://itol.embl.de/ (accessed on 10 March 2023)) [33]. Furthermore, the exon–intron organization of these m6A-related genes was obtained using TBtools [34] and visualized using the GSDS tool (http://gsds.gao-lab.org/ (accessed on 10 March 2023)) based on the wheat genome’s gff3 annotation information. Additionally, the conserved protein motif was predicted with MEME online tool (http://alternate.meme-suite.org/tools/meme (accessed on 10 March 2023)) with the maximum motif set to 20. The chromosomal location information of the m6A gene was obtained based on the genome annotation information and the BLASTN results. TBtools software was used to demonstrate the physical location of m6A on chromosomes. All upstream 2000 bp of the TSS (transcription start site) of the identified m6A-related genes were extracted as the putative promoter sequences, and the cis-acting elements were predicted using the PlantCARE online database (https://bioinformatics.psb.ugent.be/webtools/plantcare/html/ (accessed on 10 March 2023)).

### 4.3. Expression Profiles of m6A-Related Gene under Salt and Drought Stress in Aegilops tauschii

Twelve RNA-seq samples were downloaded from the NCBI Sequence Read Archive (SRA) database (https://www.ncbi.nlm.nih.gov/sra (accessed on 10 March 2023)) (Table S7) under the BioProject accession number RRJNA815810. These samples comprised of the young leaves of Aegilops tauschii subjected to salt stress (treated in NaCl solution for 5 days; SRR19659697, SRR19659698, SRR19659722), hypertonic stress (treated in PEG-6000 solution for 2 days; SRR19659694, SRR19659695, SRR19659696) and as control conditions, treated with 1/2 Hoagland solution for 2 days (SRR19659703, SRR19659704, SRR19659705) and 5 days (SRR19659699, SRR19659700, SRR19659702). Using FastQC [35] and Trimmomatic [36] software to evaluate the quality of the above sequences and filter low-quality sequences. The filtered reads were aligned to the *Aegilops tauschii* genome using the hisat2 software [37], then featureCounts program was used to perform the gene expression quantification and calculate the TPM values [38]. The heatmap of the expression patterns was made using R software. Finally, GO enrichment analysis of them was conducted using the DAVID (https://david.ncifcrf.gov/ (accessed on 10 March 2023)) [39].

### 4.4. Genetic Variation and Haplotype Frequency of m6A-Related Genes

Nucleotide variation data from 154 Triticum and Aegilops accessions were extracted from 414 Triticum accessions [40], including 99 landrace accessions (T. aestivum), 25 cultivar accessions (*T. aestivum*) and 30 *Ae. tauschii* accessions (*Aegilops tauschii* Coss. ssp. strangulate, 9; *Aegilops tauschii* Coss. ssp. tauschii var. meyeri, 11; *Aegilops tauschii* Coss. ssp. tauschii var. anathera, 10). SNPs in the coding region of m6A genes were extracted using VCFtools (version 0.1.16) [41]. Nucleotide diversity (π), fixation index (Fst) and Tajimas’D were calculated using VCFtools (version 0.1.16) with a sliding window size of 100 Kb. Furthermore, the haplotype of each m6A gene within different populations and the frequency of each type were investigated and displayed by DNAsp 6.0 software (http://www.ub.edu/dnasp (accessed on 10 March 2023)) [42], and the haplotype found in more than half of the total accessions in a population was considered as the main haplotype for the population.

### 4.5. qPCR Validation of the Selected TdNAC Genes under Salt Stress

To validate the salt-responsive m6A genes, 5 candidates obtained by RNA-seq analysis were randomly selected to investigate their expression patterns under salt stress using qRT-PCR method. Seeds of wild emmer wheat genotype As2389 were hydroponic cultured in the growth chamber under controlled conditions (22 ± 1 °C, 16 h light/8 h dark cycle). The three-week-old seedlings were subjected to 200 mM NaCl solution, and samples were collected after 0 h, 3 h, 6 h, 12h and 24 h treatment, respectively. Leaves of all samples were collected from three to five plants at each time point with three biological replications. All samples were stored at −80 °C for RNA extraction using RNA Easy Fast Plant Tissue Kit (Tiangen, Beijing, China). cDNA was synthesized using RT Master Mix Perfect Real-Time kit (Takara, Dalian, China), and qRT-PCR reactions were performed using SYBR^®^ Green Premix Pro Taq HS qPCR Kit (Ac-curate Biology, Changsha, China) according to the manufacturer’s protocol. qPCR was performed on the QuantStudioTM 7 Flex System (Thermo Fisher Scientific, Waltham, MA, USA) with the thermal cycling condition was 95 °C for 30 s followed by 40 cycles of 95 °C for 5 s, 58 °C for 34 s. TaELF-1 was used as the internal reference, and the primers used in this study were listed in Table S8. The relative expression levels were determined by 2^−ΔΔCt^ method.

## 5. Conclusions

In this study, we identified 25 m6A-related genes in *Aegilops tauschii* and categorized them into 3 subfamilies, including writer, reader and eraser. Members in the same subfamily shared similar gene structure and conserved motif composition. A lot of cis-acting elements in response to plant hormones, regulation of plant growth and development as well as biotic and abiotic stresses were found in the promoters of these Aetm6As. Furthermore, the expression patterns and co-expression network of Aetm6As under salt and drought stress were identified based on RNA-seq data, and five of them were selected to validate by qRT-PCR analysis. Finally, genetic variations of Aetm6As were detected based on public resequencing. The results demonstrate that an obvious genetic bottleneck has occurred at Aetm6As during the evolutionary process of *Aegilops tauschii* to bread wheat. This study not only shed light on the potential function of the Aetm6As family in regulating *Aegilops tauschii* and salt stress, but also provided some clues for the evolution of this family in *Aegilops tauschii* and other plants.

## Figures and Tables

**Table 1:** Genomic information and characterization of protein encoded by m6A gene family members in *Aegilops tauschii*.

Name	Gene ID	Num of Exon	Protein Length(aa)	MW (kDa)	PI	Subcellular Localization	Type
AeCPSF30-7D	AET7Gv21131400.3	7	650	71.33	6.42	Nucleus.	Reader
AeVIR-7D	AET7Gv20860000.14	28	2241	246.61	5.35	Nucleus.	Writer
AeECT1-7D	AET7Gv20706100.6	8	617	68.51	5.6	Nucleus.	Reader
AeECT2-7D	AET7Gv20672400.4	9	711	79.45	8.69	Nucleus.	Reader
AeALKBH1-7D	AET7Gv20212500.2	6	499	55.43	7.16	Nucleus.	Eraser
AeALKBH2-6D	AET6Gv20822600.5	4	267	29.27	5.11	Chloroplast.	Eraser
AeMTA-6D	AET6Gv20657900.1	7	732	81.00	7.06	Nucleus.	Writer
AeALKBH1-5D	AET5Gv21090400.1	4	438	47.73	8.77	Nucleus.	Eraser
AeALKBH2-5D	AET5Gv21090100.3	7	655	71.69	9.18	Chloroplast.	Eraser
AeECT1-5D	AET5Gv21000000.1	8	578	63.18	8.23	Nucleus.	Reader
AeECT2-5D	AET5Gv20999900.1	7	578	63.44	6.28	Nucleus.	Reader
AeECT3-5D	AET5Gv20901300.2	9	709	76.64	8.86	Nucleus.	Reader
AeMTB-4D	AET4Gv20747800.1	7	843	93.64	6.82	Nucleus.	Writer
AeECT1-4D	AET4Gv20670400.6	9	737	79.65	8.45	Nucleus.	Reader
AeECT2-4D	AET4Gv20470400.12	6	714	76.95	5.76	Nucleus.	Reader
AeECT1-3D	AET3Gv20643300.3	9	688	75.08	5.74	Nucleus.	Reader
AeECT2-3D	AET3Gv20442400.10	6	714	78.83	7.02	Nucleus.	Reader
AeALKBH1-3D	AET3Gv20211200.1	8	296	32.89	6.71	Chloroplast.	Eraser
AeALKBH2-3D	AET3Gv20038200.4	7	365	40.71	7.06	Cytoplasm.	Eraser
AeALKBH3-2D	AET2Gv20961100.2	6	348	37.76	8.52	Chloroplast.	Eraser
AeECT-2D	AET2Gv20023000.1	8	710	79.95	6.51	Nucleus.	Reader
AeHAKAI1-1D	AET1Gv20423200.1	3	423	46.71	7.33	Nucleus.	Writer
AeHAKAI2-1D	AET1Gv20394200.1	3	467	50.50	8.05	Nucleus.	Writer
AeECT1-1D	AET1Gv20267900.7	7	742	82.17	9.1	Nucleus.	Reader
AeECT2-1D	AET1Gv20103100.6	11	642	70.52	5.34	Nucleus.	Reader

**Table 2:** Composition and abundance of M6A genes in different plant species.

Species	M6a Writers					M6a Erasers	M6a Readers			
	MTs *	VIRs	FIP37s	HAKAIs	TRMs	ALKBHs	ECTs	CPSF30s	YTH	HNRNPs
*Arabidopsis thaliana*	2	2	2	2	2	14	11	1	1	
*Aegilops tauschii*	2	1		2		7	12	1		
*Triticum aestivum*	9	3	4	4		29	36	5		
*Oryza sativa*	2	1	1	1	2	14	11	1	1	
*Solanum lycopersicum*	4	1	1	1		8		2	7	
*Poplar 84K*	8	2	2	2		4	28	5		
*Beta vulgaris*	2	1	1	1		14	3	3		
*Zea mays*	3	2	5	2		10	25	1		
*Marchantia polymorpha*	3	2	1	1		10	4	2		
*Camellia sinensis*	5	2	1	1		16	8	1		
*Vitis vinifera*	3	1	1	1		11	11	2		
*Gossypium hirsutum*	5	3	2	5		26	29			
*Physcomitrella patens*	4	2	2	3		7	4			
*Litchi chinensis Sonn.*	2	1	4			12	8	2		2
*Selaginella moellendorffii*	2		1			16	4	2		

* MTs includes MTA, MTB and MTC.

## Data Availability

All of the datasets supporting the results of this article are included within the article and its Supplementary Materials.

## Supplementary Materials

The following supporting information can be downloaded at: https://www.mdpi.com/article/10.3390/plants12142747/s1, Figure S1: GO enrichment analysis of differentially expressed m6A gene in *Aegilops tauschii*; Table S1: The cis-elements identified in the promoter regions of the 64 m6A-related genes in wild emmer wheat; Table S2: The TPM value of each m6-related genes at different time points under salt stress; Table S3: GO enrichment analysis of m6A genes; Table S4: Tajiman’D of m6a genes in tauschii, landrace and cultivar; Table S5: Fst and π of m6a genes in tauschii, landrace and cultivar; Table S6: Haplotype of m6a genes in tauschii, landrace and cultivar; Table S7: The detailed information of the RNA-seq dataset used in this study; Table S8: Primers used for qPCR in this study.
