# Supplementary figures and images for: Genome-Wide Identification, Expression and Evolution Analysis of m6A Writers, Readers and Erasers in Aegilops_tauschii
Source: Plants (Basel). 2023 Jul 24;12(14):2747. doi: 10.3390/plants12142747 (PMC10385245; doi:10.3390/plants12142747)

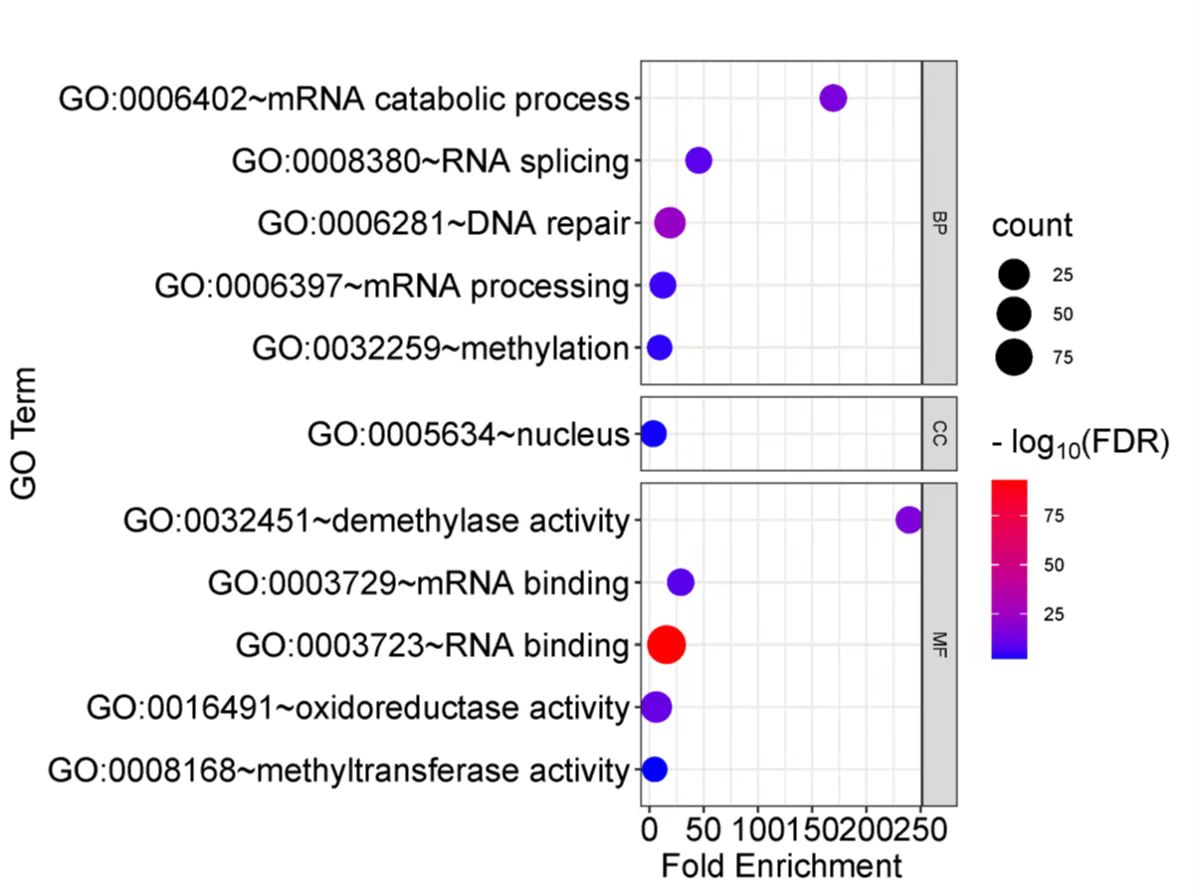

Supplement: Supplementary file 1 [file plants-12-02747-s001.zip › Figure S1.jpg]
